# Supplementary material for: Satellite record reveals 1960s acceleration of Totten Ice Shelf in East Antarctica
Source: Nat Commun. 2023 Jul 10;14:4061. doi: 10.1038/s41467-023-39588-x (PMC10333183; doi:10.1038/s41467-023-39588-x)
Supplement: Supplementary file 1 — Supplementary Information [file 41467_2023_39588_MOESM1_ESM.pdf]

**Supplementary Information for**

# Satellite record reveals 1960s acceleration of Totten Ice Shelf, East Antarctica

Rongxing Li[1, 2], Yuan Cheng[3, a], Tian Chang[1, 2], David E. Gwyther[4], Martin Forbes[5], Lu An[1, 2, b], Menglian Xia[1, 2], Xiaohan Yuan[1, 2], Gang Qiao[1, 2], Xiaohua Tong[1, 2], and Wenkai Ye[1, 2]

[1]Center for Spatial Information Science and Sustainable Development Applications, Tongji University, 1239 Siping Road, Shanghai, China; [2]College of Surveying and Geo-Informatics, Tongji University, 1239 Siping Road, Shanghai, China; [3]Institute for the Conservation of Cultural Heritage, School of Cultural Heritage and Information Management, Shanghai University, Shanghai, China; [4]School of Earth and Environmental Sciences, The University of Queensland, St Lucia, QLD, 4072, Australia; [5]National School of Surveying, University of Otago, Dunedin, New Zealand

[a, b] To whom correspondence should be addressed. Email: chengyuan@shu.edu.cn, anlu2021@tongji.edu.cn

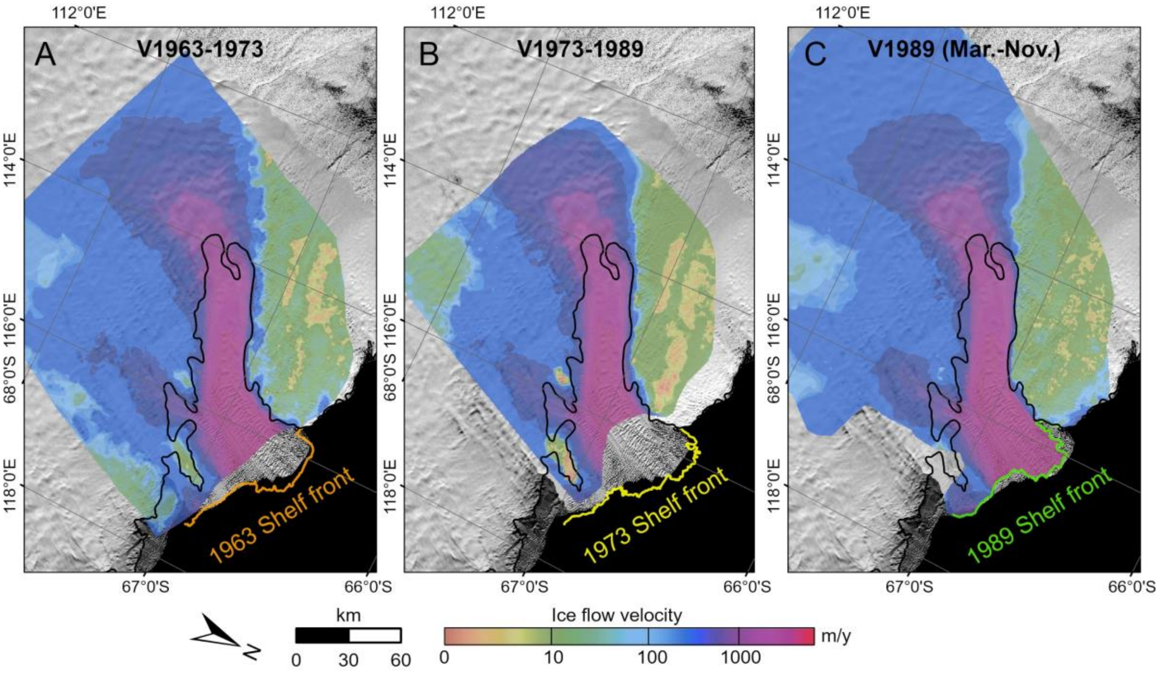


**Fig. S1.** Three velocity maps of Totten Glacier: (A) 1963–1973 map, (B) 1973–1989 map, and (C) 1989 (March–November) map. The grounding line (black) is from ref. [1]. Background images are from the LIMA mosaic[2].

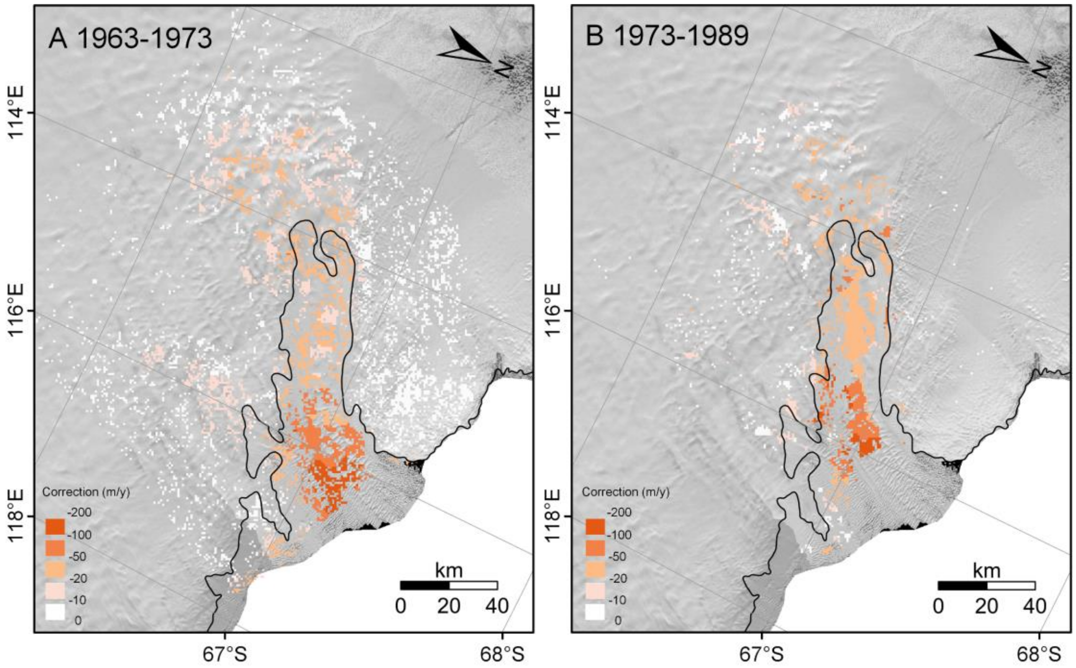


**Fig. S2.** Velocity overestimation corrections applied to velocity maps with long timespans: (A) 1963–1973 map and (B) 1973–1989 map. The grounding line (black) is from ref. [1]. Background images are from the LIMA mosaic[2].

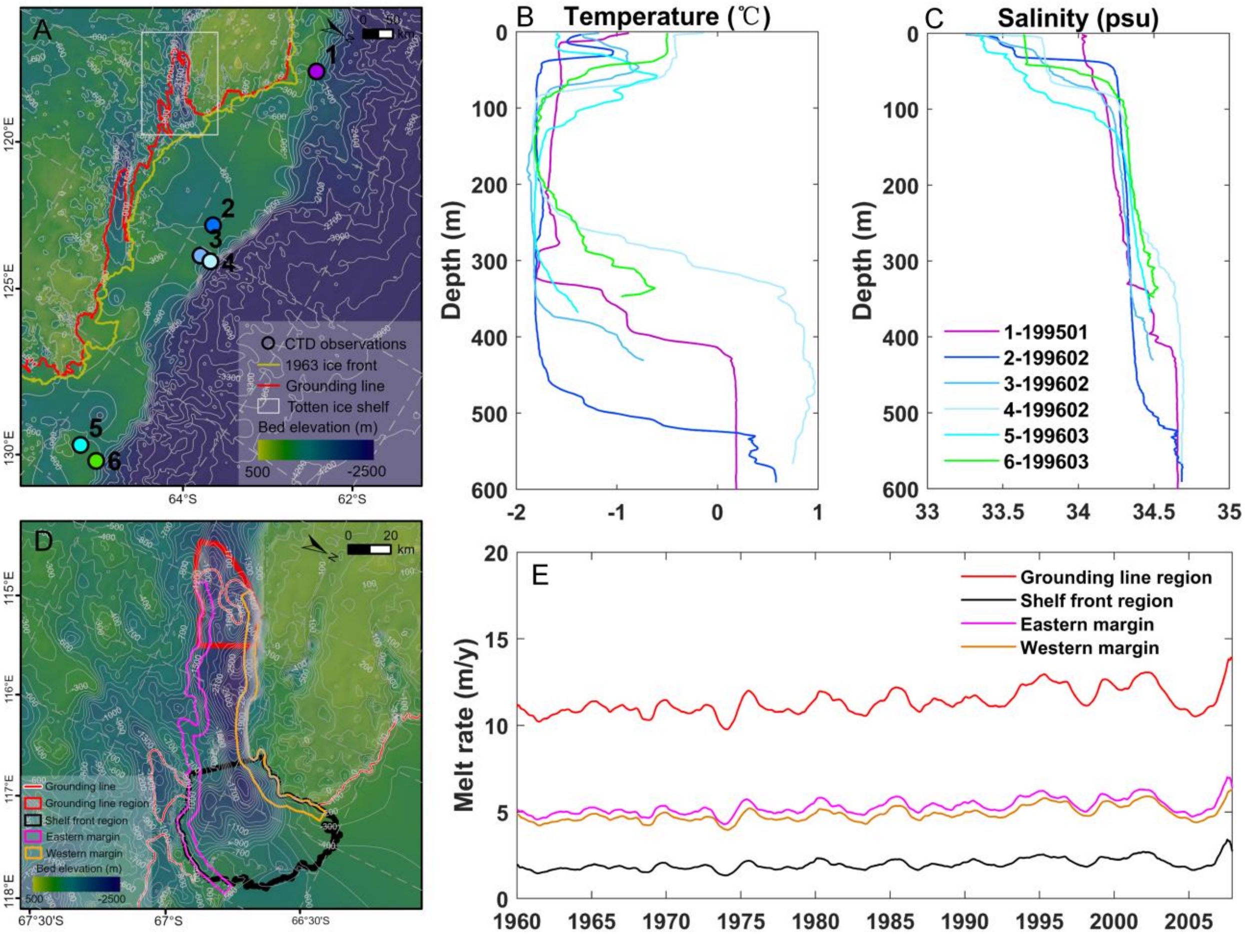


**Fig. S3.** (A) Locations of six profiles of CTD observations collected in austral summer of 1995-1996 outside the TIS shelf front and on the continent shelf and slope, from the World Ocean Database (WOD). White box indicates the extend of the TIS region in (D). Also shown are the bed topography of grounded ice and the bathymetry underneath and outside the ice shelf, from BedMachine Antarctica[3]. (B) Potential temperature ($^{o}$C) and (C) salinity in practical salinity units (PSU) of the six CTD profiles for the locations shown in (A). Profile labels are formatted as Profile ID-YearMonth. (D) Enlarged area of TIS with bathymetry and boundaries of regions for presenting basal melting modelling results in (E). (E) Modelled melt rates from 1960 to 2007 in the grounding line region, eastern and western margins, and shelf front region. The grounding line is from ref. [1].

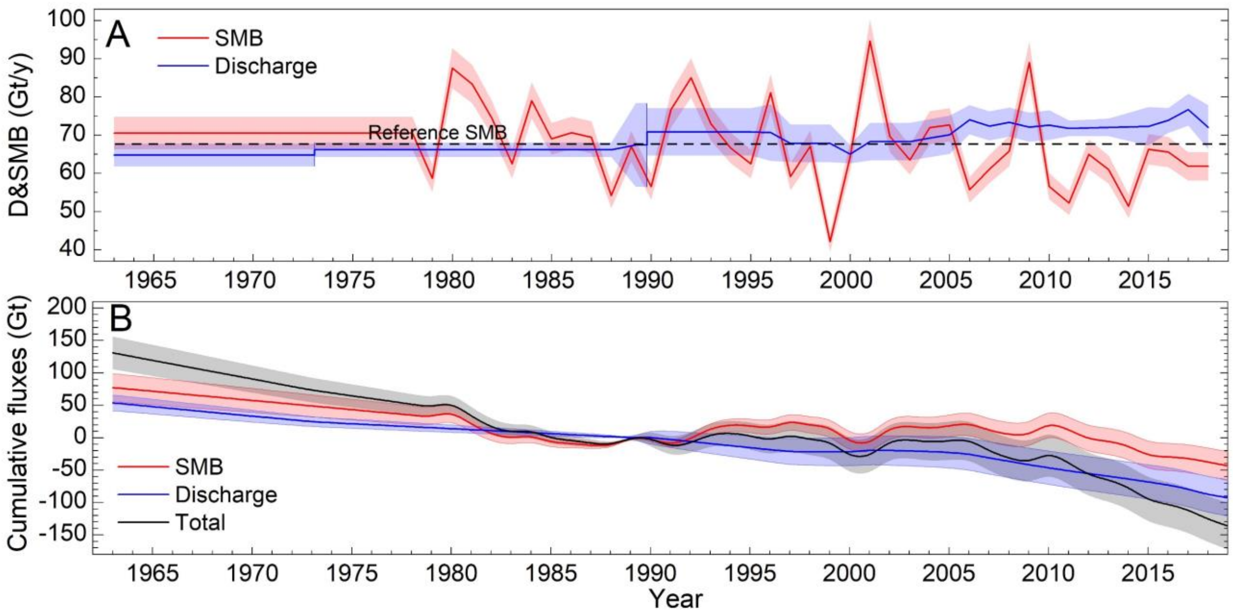


**Fig. S4.** (A) Long-term (1963–2018) SMB (RACMO 2.3 p2) and ice discharge derived from the 1963–1989 maps from this study and 1989–2018 maps from refs. [4–6]. (B) Cumulative SMB, discharge, and mass balance are computed with the transition point of 1989 as the starting time for forward and backward cumulative mass change integration of each item minus reference SMB. The MB in the TG basin from 1963 to 2018 is dominated by long-term accelerated ice discharge, modulated by the SMB.

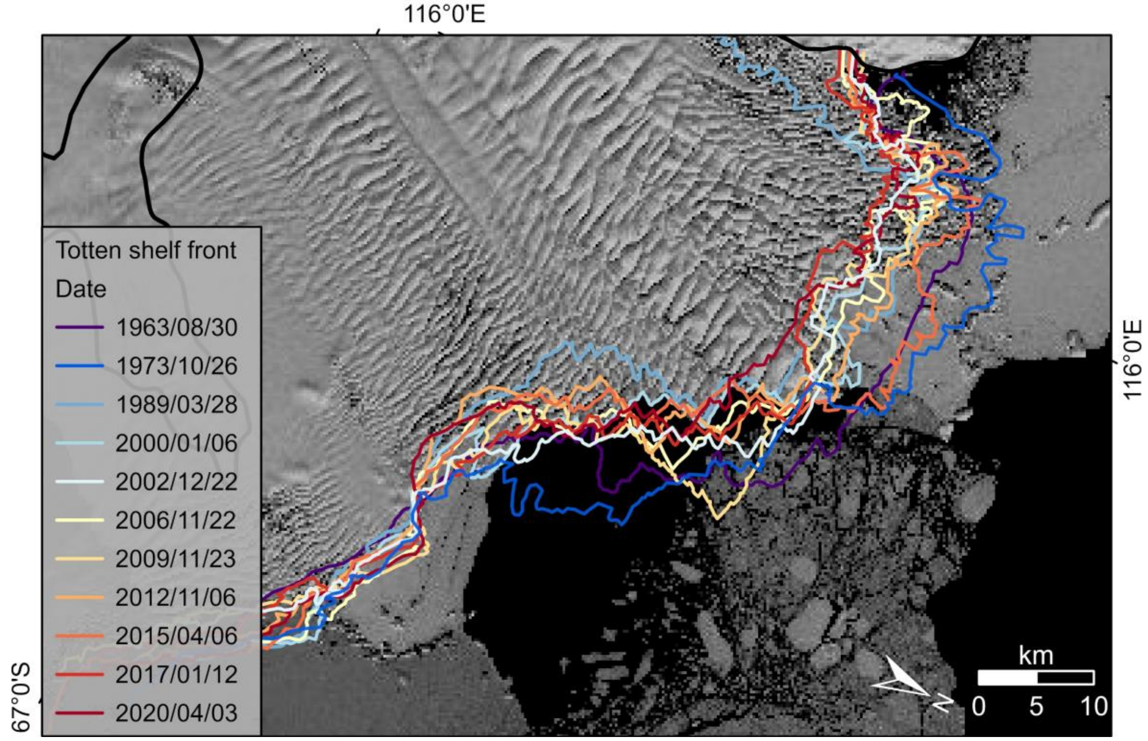


**Fig. S5.** From 1963 to 2020, the TIS front has advanced and retreated within a range of ~15 km. The ice front series are extracted from the satellite images listed in Table S3. Background image is from the LIMA mosaic[2]. The grounding line (black) is from ref. [1].

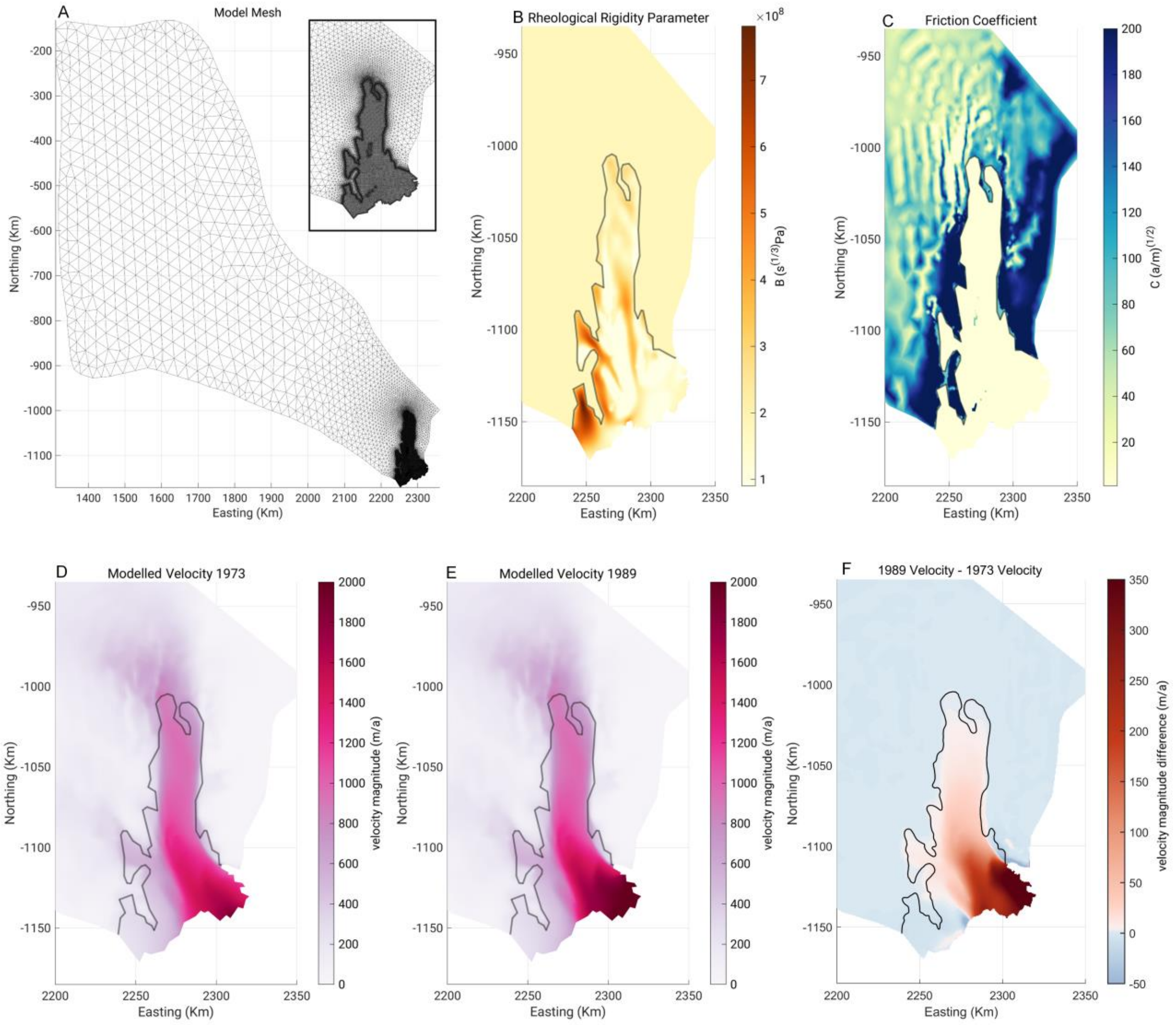


**Fig. S6.** (A) Model mesh for Totten glacier drainage basin and ice shelf using the 1973 ice shelf front. Mesh sizes range from 600 to 35000 m in the grounded area and 600 to 700 m in the ice shelf or over ice rises (see inset). (B) and (C) Spatial distribution of the ice rigidity parameter B and ISSM friction coefficient C, respectively, in the Totten ice shelf region. The grounding line is from (ref [1]) and rumples extents are simplified from (ref [7]). The ice surface and bed elevations are interpolated from Bedmachine Antarctica V3[8].The velocity field used for inversion is the 1963-73 velocity map (Fig. S1A). The outside portion on grounded ice is filled with velocity of MEaSUREs V2[9]. (D) Modelled velocity field of 1973 using 1973 shelf front. (E) Modelled velocity field of 1989 using 1989 shelf front with the large calving front retreat in the western margin. (F) Difference between the modelled velocity maps of 1989 (E) and 1973 (D). Ice shelf extent not covered by the 1963-73 velocity map (Fig. S1A) is not included in (D) to (F).

**Table S1.** Computation of uncertainties of three velocity maps (Fig. S1) using the equation in Materials and Methods.

| Ice velocity map | Sensor | $\sigma_{ortho}$ (m) | $\sigma_{ident}$ (m) | $\sigma_{match}$ (m) | $\Delta t$ (year) | $\sigma_{vel}$ (m/y) |
|---|---|---|---|---|---|---|
| 1963-1973 | ARGON | 84.7 | 118 | 60.0 | 10.2 | 16.7 |
| | MSS | 52.6 | | | | |
| 1973-1989 | MSS | 52.6 | 30.0 | 15.0 | 16.0 | 4.3 |
| | TM | 30.9 | | | | |
| 1989 | TM | 30.9 | 15.0 | 15.0 | 0.6 | 79.2 |
| | TM | 30.9 | | | | |

**Table S2.** Information for images used in velocity mapping. The images with bold IDs were used for the three ice velocity maps in Fig. S1. Other images were used for the extended map area in Fig. 1A. PCI is the Geomatica OrthoEngine software system[10].

| Image ID | Sensor | Date | Resolution (m) | Orthorectification method |
|---|---|---|---|---|
| **DS09059A037MC077** | ARGON (KH-5) | 10/29/1963 | 59 (Scanned at 33) | Bundle adjustment |
| DS09059A037MC078 | ARGON (KH-5) | 10/29/1963 | 59 (Scanned at 33) | |
| **DS09058A006MC115** | ARGON (KH-5) | 08/30/1963 | 59 (Scanned at 33) | Bundle adjustment |
| DS09058A006MC116 | ARGON (KH-5) | 08/30/1963 | 59 (Scanned at 33) | |
| LM11031091972336FAK02 | Landsat-1 (MSS) | 12/01/1972 | 60 | Toutin's model in PCI |
| LM11091071973031AAA04 | Landsat-1 (MSS) | 01/31/1973 | 60 | Toutin's model in PCI |
| **LM11071071973299AAA05** | Landsat-1 (MSS) | 10/26/1973 | 60 | Toutin's model in PCI |
| **LM11071081973299AAA05** | Landsat-1 (MSS) | 10/26/1973 | 60 | Toutin's model in PCI |
| LM11111081973321AAA05 | Landsat-1 (MSS) | 11/17/1973 | 60 | Toutin's model in PCI |
| LT41051061989044XXX02 | Landsat-4 (TM) | 02/13/1989 | 30 | Toutin's model in PCI |
| **LT41021071989087XXX12** | Landsat-4 (TM) | 03/28/1989 | 30 | Toutin's model in PCI |
| **LT41021081989087XXX12** | Landsat-4 (TM) | 03/28/1989 | 30 | Toutin's model in PCI |
| LT41021091989087XXX07 | Landsat-4 (TM) | 03/28/1989 | 30 | Toutin's model in PCI |
| LT41001081989089XXX02 | Landsat-4 (TM) | 03/30/1989 | 30 | Toutin's model in PCI |
| LT41001091989089XXX02 | Landsat-4 (TM) | 03/30/1989 | 30 | Toutin's model in PCI |
| **LT41021071989311XXX04** | Landsat-4 (TM) | 11/07/1989 | 30 | Toutin's model in PCI |
| **LT41021081989311XXX02** | Landsat-4 (TM) | 11/07/1989 | 30 | Toutin's model in PCI |
| LT41021091989311XXX02 | Landsat-4 (TM) | 11/07/1989 | 30 | Toutin's model in PCI |
| LT41051081989316XXX03 | Landsat-4 (TM) | 11/12/1989 | 30 | Toutin's model in PCI |
| LT41001091989345XXX02 | Landsat-4 (TM) | 12/11/1989 | 30 | Toutin's model in PCI |
| LT41001081989361XXX03 | Landsat-4 (TM) | 12/27/1989 | 30 | Toutin's model in PCI |

**Table S3.** Information about time of calving front (Fig. S5), image used for derivation, ice shelf area, lost area (Fig. 2B), and corresponding velocity and weight in Boxes 1 and 2 (Figs. 2A and 2C), respectively. The velocity is from this study, Mouginot et al.[4], Gardner et al.[5] and Rignot et al.[6].

| Date | Image ID | Ice shelf area ($km^2$) | Shelf area loss ($km^2$) | Velocity in Box 1 (m/y) | Weight | Velocity in Box 2 (m/y) | Weight |
|---|---|---|---|---|---|---|---|
| 1963/8/30 | Kim et al.,2007 | 5702 | | | | | |
| 1973/10/26 | LM11071071973299AAA05 | 5848 | -146 | 1328 | 0.63 | -43 | 0.63 |
| 1989/3/28 | LT41021071989087XXX12 | 5287 | 561 | 1441 | 1.00 | 52 | 1.00 |
| 2000/1/6 | LE71021072000006EDC00 | 5450 | -163 | 1257 | 0.44 | -21 | 0.44 |
| 2002/12/22 | LE71011072002356EDC00 | 5499 | -49 | 1267 | 0.13 | | |
| 2006/11/22 | LE71021072006326EDC00 | 5494 | 5 | 1325 | 0.13 | 38 | 0.13 |
| 2009/11/23 | LE71011072009327EDC00 | 5524 | -30 | 1319 | 0.06 | 52 | 0.13 |
| 2012/11/6 | LE71021072012311EDC00 | 5453 | 71 | 1307 | 0.06 | 34 | 0.13 |
| 2015/4/6 | LC81011072015096LGN01 | 5530 | -77 | 1243 | 0.06 | -24 | 0.06 |
| 2017/1/12 | LC81021072017012LGN01 | 5418 | 112 | 1266 | 0.06 | -36 | 0.06 |
| 2020/4/3 | LC81011072020094LGN00 | 5384 | 34 | 1294 | 0.06 | -51 | 0.06 |

Note: Velocity in Box 2 is recalculated by removing a long-term increase trend ($y = 2.1984x – 3606.3$)

**Table S4.** Cumulative Surface mass balance (SMB), ice discharge (D) and mass balance (MB) and their uncertainties (Gt) from 1963 to 2019 (Fig. S4B). The transition point of 1989 is used as the starting time for forward and backward cumulative mass change integration of each item minus reference SMB. All unit in Gt.

| Year | SMB | $\sigma_{SMB}$ | D | $\sigma_{D}$ | MB | $\sigma_{MB}$ |
|---|---|---|---|---|---|---|
| 1963 | 77.2 | 21.7 | 53.5 | 12.1 | 130.7 | 24.8 |
| 1964 | 74.3 | 21.3 | 50.6 | 11.7 | 124.9 | 24.3 |
| 1965 | 71.5 | 20.9 | 47.7 | 11.3 | 119.2 | 23.7 |
| 1966 | 68.7 | 20.4 | 44.8 | 10.9 | 113.4 | 23.2 |
| 1967 | 65.8 | 20.0 | 41.9 | 10.5 | 107.7 | 22.6 |
| 1968 | 63.0 | 19.5 | 39.0 | 10.1 | 102.0 | 22.0 |
| 1969 | 60.1 | 19.1 | 36.1 | 9.6 | 96.2 | 21.4 |
| 1970 | 57.3 | 18.6 | 33.1 | 9.2 | 90.5 | 20.7 |
| 1971 | 54.5 | 18.1 | 30.2 | 8.7 | 84.7 | 20.1 |
| 1972 | 51.6 | 17.6 | 27.3 | 8.1 | 79.0 | 19.4 |
| 1973 | 48.8 | 17.1 | 24.4 | 7.6 | 73.2 | 18.7 |
| 1974 | 46.0 | 16.6 | 22.9 | 7.3 | 68.8 | 18.1 |
| 1975 | 43.1 | 16.0 | 21.3 | 7.1 | 64.5 | 17.5 |
| 1976 | 40.3 | 15.4 | 19.8 | 6.8 | 60.1 | 16.9 |
| 1977 | 37.5 | 14.8 | 18.3 | 6.6 | 55.8 | 16.2 |
| 1978 | 34.6 | 14.2 | 16.8 | 6.3 | 51.4 | 15.6 |
| 1979 | 31.8 | 13.6 | 15.2 | 6.0 | 47.1 | 14.8 |
| 1980 | 40.8 | 13.1 | 13.7 | 5.7 | 54.5 | 14.3 |
| 1981 | 21.0 | 12.0 | 12.2 | 5.4 | 33.2 | 13.2 |
| 1982 | 5.3 | 10.9 | 10.7 | 5.0 | 16.0 | 12.0 |
| 1983 | -1.3 | 10.0 | 9.1 | 4.6 | 7.8 | 11.0 |
| 1984 | 3.9 | 9.2 | 7.6 | 4.2 | 11.5 | 10.2 |
| 1985 | -7.5 | 7.9 | 6.1 | 3.8 | -1.4 | 8.8 |
| 1986 | -8.8 | 6.8 | 4.6 | 3.3 | -4.3 | 7.5 |
| 1987 | -11.8 | 5.3 | 3.0 | 2.7 | -8.7 | 5.9 |
| 1988 | -13.5 | 3.3 | 1.5 | 1.9 | -12.0 | 3.8 |
| 1989 | 0.0 | 0.0 | 0.0 | 0.0 | 0.0 | 0.0 |
| 1990 | -0.6 | 4.0 | 0.3 | 10.9 | -0.4 | 11.6 |
| 1991 | -11.8 | 5.3 | -2.9 | 12.6 | -14.7 | 13.6 |
| 1992 | -2.8 | 7.0 | -6.1 | 14.0 | -8.8 | 15.7 |
| 1993 | 14.5 | 8.7 | -9.2 | 15.3 | 5.3 | 17.6 |
| 1994 | 19.8 | 9.7 | -12.4 | 16.5 | 7.4 | 19.2 |
| 1995 | 18.7 | 10.5 | -15.5 | 17.7 | 3.2 | 20.5 |
| 1996 | 13.5 | 11.1 | -18.7 | 18.7 | -5.2 | 21.8 |
| 1997 | 26.9 | 12.2 | -21.7 | 20.1 | 5.2 | 23.5 |
| 1998 | 18.4 | 12.7 | -21.9 | 20.7 | -3.5 | 24.3 |
| 1999 | 17.8 | 13.3 | -22.0 | 21.3 | -4.2 | 25.1 |
| 2000 | -7.7 | 13.5 | -22.2 | 21.8 | -29.9 | 25.7 |
| 2001 | -11.0 | 14.1 | -19.5 | 22.0 | -30.5 | 26.1 |
| 2002 | 15.8 | 15.2 | -20.1 | 22.5 | -4.3 | 27.1 |
| 2003 | 17.9 | 15.7 | -20.7 | 23.0 | -2.8 | 27.9 |
| 2004 | 13.7 | 16.2 | -21.3 | 23.6 | -7.6 | 28.6 |
| 2005 | 18.0 | 16.8 | -22.8 | 24.1 | -4.9 | 29.3 |
| 2006 | 23.0 | 17.3 | -25.2 | 24.6 | -2.3 | 30.1 |
| 2007 | 11.0 | 17.6 | -31.6 | 24.9 | -20.6 | 30.5 |
| 2008 | 4.4 | 18.0 | -36.3 | 25.2 | -31.8 | 30.9 |
| 2009 | 2.6 | 18.4 | -42.0 | 25.4 | -39.4 | 31.4 |
| 2010 | 23.9 | 19.2 | -46.4 | 25.7 | -22.5 | 32.1 |
| 2011 | 12.8 | 19.5 | -51.3 | 26.0 | -38.4 | 32.5 |
| 2012 | -2.6 | 19.7 | -55.4 | 26.1 | -58.0 | 32.7 |
| 2013 | -5.3 | 20.1 | -59.6 | 26.2 | -64.9 | 33.0 |
| 2014 | -12.1 | 20.5 | -63.9 | 26.3 | -76.0 | 33.3 |
| 2015 | -28.4 | 20.7 | -68.4 | 26.5 | -96.8 | 33.7 |
| 2016 | -29.8 | 21.1 | -73.0 | 27.0 | -102.8 | 34.3 |
| 2017 | -31.9 | 21.4 | -79.2 | 27.2 | -111.1 | 34.6 |
| 2018 | -37.8 | 21.7 | -88.1 | 27.5 | -125.9 | 35.1 |

| | | | | | | |
|---|---|---|---|---|---|---|
| 2019 | -43.7 | 22.1 | -92.5 | 28.1 | -136.2 | 35.7 |

**Supplementary References**


1. Rignot, E., Mouginot, J. & Scheuchl, B. Antarctic grounding line mapping from differential satellite radar interferometry. Geophys. Res. Lett. 38, L10504 (2011).
2. Bindschadler, R. et al. The Landsat image mosaic of Antarctica. Remote Sens. Environ. 112, 4214–4226 (2008).
3. Morlighem, M. et al. Deep glacial troughs and stabilizing ridges unveiled beneath the margins of the Antarctic ice sheet. Nat. Geosci. 13, 132–137 (2020).
4. Mouginot, J., Scheuchl, B. & Rignot, E. MEaSUREs Annual Antarctic Ice Velocity Maps, Version 1. (2017) doi:10.5067/9T4EPQXTJYW9.
5. Gardner, A. S., Fahnestock, M. A. & Scambos, T. A. MEaSUREs ITS_LIVE Landsat Image-Pair Glacier and Ice Sheet Surface Velocities: Version 1. (2019) doi:10.5067/IMR9D3PEI28U.
6. Rignot, E., Scheuchl, B. & Mouginot, J. MEaSUREs Multi-year Reference Velocity Maps of the Antarctic Ice Sheet, Version 1. (2022) doi:10.5067/FB851ZIZYX5O.
7. Mouginot, J., Scheuchl, B. & Rignot, E. MEaSURES Antarctic Boundaries for IPY 2007-2009 from Satellite Radar, Version 2. (2017) doi:10.5067/AXE4121732AD.
8. Morlighem, M. MEaSUREs BedMachine Antarctica, Version 3. (2022) doi:10.5067/FPSU0V1MWUB6.
9. Rignot, E., Mouginot, J. & Scheuchl, B. MEaSUREs InSAR-Based Antarctica Ice Velocity Map, Version 2. (2017) doi:10.5067/D7GK8F5J8M8R.
10. PCI Geomatica User Guide. (2005).
